# Supplementary material for: Unsupervised learning architecture for classifying the transient noise of interferometric gravitational-wave detectors
Source: Sci Rep. 2022 Jun 15;12:9935. doi: 10.1038/s41598-022-13329-4 (PMC9200730; doi:10.1038/s41598-022-13329-4)
Supplement: Supplementary file 1 — Supplementary Information. [file 41598_2022_13329_MOESM1_ESM.pdf]

Supplemental Material: Unsupervised Learning Architecture for Classifying the Transient Noise of Interferometric Gravitational-wave Detectors

Yusuke Sakai[1,+], Yousuke Itoh[2, 3], Piljong Jung[4], Keiko Kokeyama[5], Chihiro Kozakai[6], Katsuko T. Nakahira[7], Shoichi Oshino[8], Yutaka Shikano[9, 10, 11], Hirotaka Takahashi[1, 12, 13, ++], Takashi Uchiyama[8], Gen Ueshima[7], Tatsuki Washimi[6], Takahiro Yamamoto[8], and Takaaki Yokozawa[8]

[1]Research Center for Space Science, Advanced Research Laboratories, Tokyo City University, Setagaya-ku, Tokyo 158-0082, Japan
[2]Graduate School of Science, Osaka Metropolitan University, Sumiyoshi-ku, Osaka City, Osaka 558-8585, Japan
[3]Nambu Yoichiro Institute of Theoretical and Experimental Physics (NITEP), Osaka Metropolitan University,, Sumiyoshi-ku, Osaka City, Osaka 558-8585, Japan
[4]National Institute for Mathematical Sciences, Daejeon 34047, Republic of Korea
[5]School of Physics and Astronomy, Cardiff University, The Parade, Cardiff, CF24 3AA United Kingdom
[6]Gravitational Wave Science Project, Kamioka branch, National Astronomical Observatory of Japan, Hida City, Gifu 506-1205, Japan
[7]Department of Information and Management Systems Engineering, Nagaoka University of Technology, Nagaoka, Niigata 940-2188, Japan
[8]Institute for Cosmic Ray Research, KAGRA Observatory, The University of Tokyo, Hida City, Gifu 506-1205, Japan
[9]Graduate School of Science and Technology, Gunma University, Maebashi, Gunma 371-8510, Japan
[10]Institute for Quantum Studies, Chapman University, Orange, CA 92866, USA
[11]JST PRESTO, Kawaguchi, Saitama 332-0012, Japan
[12]Institute for Cosmic Ray Research, The University of Tokyo, Kashiwa City, Chiba 277-8582, Japan
[13]Earthquake Research Institute, The University of Tokyo, Bunkyo-ku, Tokyo 113-0032, Japan
[+]g2191402@tcu.ac.jp
[++]hirotaka@tcu.ac.jp

ABSTRACT

A comparison of the classification results shown in Figure 4 of the main text with the feature visualisation using t-SNE is discussed in this supplemental material.

Feature Visualization of Transient Noise using t-SNE

Generally, the feature space for the input data is high-dimensional, and it can be expressed visually by reducing it to a low-dimensional space. One of the nonlinear dimensionality reductions[1] is the t-distributed stochastic neighbour embedding (t-SNE)[2]. The t-SNE algorithm embeds the data in high-dimensional space as a t-SNE component in low-dimensional space, and this embedding is effective for visualising the data in a high-dimensional space.

In this study, a composition mapping $h \circ f : \mathbb{R}^{224\times224\times4} \to \mathbb{R}^2$ is used to evaluate the features of the transient noise, where $f : \mathbb{R}^{224\times224\times4} \to \mathbb{R}^{512}$ is a mapping from the input space to the feature space using a pre-trained encoder of the VAE and $h : \mathbb{R}^{512} \to \mathbb{R}^2$ is an embedding from the feature space to a 2D plane using t-SNE. The feature visualisation of transient noise is shown at the left of Figure S1, and the parameters used for the embedding are shown at the bottom right of Figure S1.

We investigated how the Gravity Spy dataset is clustered in the feature space by visualisation using t-SNE. Considering the data on the Gravity Spy labels of “1080Lines”, “Chirp”, “Helix”, “Scratchy”, and “Tomte”, it can be observed that they are formed closely on the feature visualisation. Therefore, the features of the transient noise by unsupervised learning are consistent with the Gravity Spy classes. Regarding the data of “Power_Line”, “Extremely_Loud”, “Scattered_Light”, and “Violin_Mode”, these distributions are separated on feature visualisation. Similar results for the separation of these data have been obtained in the unsupervised classification (see Figure 4 of the main text). Considering the data of “None_of_the_Above”,

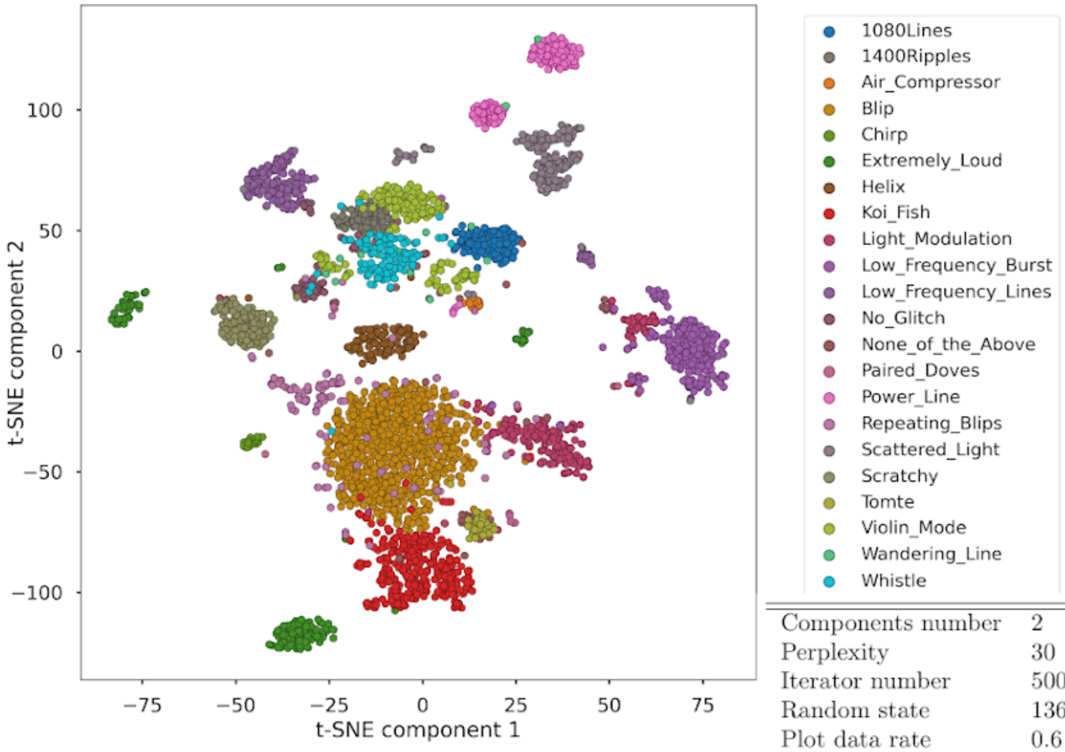

Components number	2
Perplexity	30
Iterator number	5000
Random state	136
Plot data rate	0.6

Figure S1. Left: Visualisation of the feature of transient noise. The visualisation is computed by a composition mapping $(h \circ f)$, where f is the pre-trained encoder of the VAE, which is a mapping from the input space $\mathbb{R}^{224\times224\times4}$ to the feature space $\mathbb{R}^{512}$. h is t-SNE, which is an embedding from the feature space $\mathbb{R}^{512}$ to the 2D plane. By using the Gravity Spy labels for the input data, the embedding data have labels that are useful for visualisation on the plane. Top right: List of colours corresponding to the Gravity Spy labels. Bottom right: embedding parameters used for the t-SNE. The number of components indicates the number of dimensions after the embedding. Perplexity is a characteristic that is related to the number of nearest neighbours, and the iterator number refers to the maximum number of optimisations for exploring the nearest neighbours. The plotted data rate is the ratio of the number of plots to the entire dataset.

which are not classified as a unique class on the confusion matrix shown in Figure 4 of the main text, it can be confirmed that they are not formed closely, even on the feature visualisation. Therefore, consistent results on the above labels/classes were obtained between the feature visualisation and the result of unsupervised classification.

On the contrary, considering the data of “Blip”, “Koi_Fish” are separated into subclasses on the confusion matrix shown in Figure 4 of the main text, whereas “Blip” and “Koi_Fish” are formed as one distribution, respectively, on feature visualisation in Figure S1. This formation seems to have resulted in the superposition of small classes by embedding the data into a 2D plane. Because the distribution of “Blip” and “Koi_Fish” must be separated at higher dimensions, the result of the unsupervised learning shown in Figure 4 of the main text implies that “Blip” and “Koi_Fish” have subclasses.

References

1. Roweis, S. T. & Saul, L. K. Nonlinear dimensionality reduction by locally linear embedding. *Science* **290**, 2323–2326, DOI: 10.1126/science.290.5500.2323 (2000).

2. Van der Maaten, L. & Hinton, G. Visualizing data using t-SNE. *J. machine learning research* **9**, 2579–2605 (2008).
